# Instability of Global Burden of Disease Estimates of Deaths and Disability-Adjusted Life-Years From Major Risk Factors: A Meta-Epidemiological Analysis

**DOI:** 10.1001/jamahealthforum.2026.0108

**Published:** 2026-03-13

**Authors:** Emmanuel A. Zavalis, Angelo Maria Pezzullo, John P. A. Ioannidis

**Affiliations:** 1 Department of Physiology and Pharmacology, Karolinska Institutet, Stockholm, Sweden; 2 Department of Medicine Huddinge, Karolinska Institutet, Stockholm, Sweden; 3 Meta-Research Innovation Center at Stanford, Stanford University, Stanford, California; 4 Section of Hygiene, Department of Life Sciences and Public Health, Università Cattolica del Sacro Cuore, Rome, Italy; 5 Department of Medicine, Stanford University, Stanford, California; 6 Department of Epidemiology and Population Health, Stanford University, Stanford, California; 7 Department of Biomedical Data Science, Stanford University, Stanford, California

## Abstract

**Importance:**

The Global Burden of Disease (GBD) reports widely used estimates of mortality and disability-adjusted life-years (DALYs) and related risk factors. However, the overall reliability of these estimates between GBD iterations has not been assessed.

**Objective:**

To evaluate the instability and inconsistency of GBD risk factor estimates for mortality and DALYs across GBD iterations.

**Data Sources:**

GBD risk factor collaboration estimates extracted from the published tables of GBD iterations and the Institute for Health Metrics and Evaluation repository.

**Study Selection:**

GBD risk factor collaboration publications published for 2010 through 2023.

**Data Extraction and Synthesis:**

Death and DALY estimates were manually extracted by 1 reviewer with independent validation of a random sample of 100 by another with no discrepancies. Risk factor naming was harmonized across iterations to ensure comparability; those with inconsistent definitions were excluded.

**Main Outcomes and Measures:**

Fluctuations were calculated for numbers of deaths and DALYs for each risk factor across GBD iterations during the study period (2010-2023) and between the original and subsequently revised estimates for each year (1990-2021). Differences were expressed as a ratio of the minimum to maximum range to the mean (R:M) and coefficient of variation. Detail analyses assessed diet and low physical activity. Point estimates were compared to the previous iterations’ estimates 95% uncertainty intervals (95% UI) for GBD 2019, 2021, and 2023.

**Results:**

Across GBD iterations from 2010 to 2023, the median (range) R:M was 0.8 (0-3.8) for deaths, and 0.7 (0.1-3.3) for DALYs. Level 2 dietary and child and maternal malnutrition death estimates showed high instability (R:M >1 for 9 of 16 and 4 of 8 risks, respectively). When comparing original estimates with GBD 2019, 2021, and 2023 estimates for the same years, the median R:M was 0.4 (0-2.9) for both deaths and DALYs. The coefficient of variation was greater than 0.2 for 336 of 675 death estimates (50%). Specifically, 70% to 96% of point estimates for red meat, sugar-sweetened beverages, fruits, vegetables, and seafood omega-3 fatty acids in GBD 2021 fell outside the GBD 2019 95% UI. In GBD 2023, only diet high in trans fats had more than half of point estimates outside the GBD 2021 95% UI.

**Conclusions and Relevance:**

This meta-epidemiological assessment indicates that GBD estimates are substantially unstable, particularly for behavioral risks, making them unlikely to simply reflect genuine changes over time, and warranting caution in interpretation.

## Key Points

**Question:**

How stable and consistent are the Global Burden of Disease (GBD) estimates for mortality and disability-adjusted life-years (DALYs) that are attributable to major risk factors from 2010 to 2023?

**Findings:**

This meta-epidemiological assessment comparing estimates across 8 iterations of GBD found substantial instability in behavioral risk, particularly dietary risks. Comparing revised estimates across iterations, half of the estimates had a coefficient of variation exceeding 0.2, and one-third of estimates for dietary risks in GBD 2021 fell outside the corresponding GBD 2019 uncertainty intervals.

**Meaning:**

GBD risk factor estimates, especially for behavioral and dietary risks, show marked inconsistency likely reflecting methodologic or data changes rather than true burden shifts.

## Teaser

This meta-epidemiological assessment evaluates the reliability of the Global Burden of Disease risk factor estimates for mortality and disability-adjusted life-years.

## Introduction

The first Global Burden of Disease (GBD) estimates for diseases and risk factors were produced more than 3 decades ago.^1^ They have been updated regularly, most recently in 2025 with estimates through 2023.^2^ Other institutions (eg, the United Nations International Children’s Emergency Fund, the World Health Organization, the Non-Communicable Diseases Risk Factor Collaboration) also produce global descriptive health estimates. However, the GBD’s scope,^3^ citation impact, and health policy bearing is unparalleled. GBD estimates are not just useful for supporting citations, but also likely influence priority lists for research funding^4^ and shape health policy.^5,6^ GBD uses a complex methodologic machinery and diverse sources of data (eFigure 1 in Supplement 1). Both the methodologic machinery and the data sources and processing have been amended over time (eFigure 2 in Supplement 1). Each GBD iteration re-estimates the entire time series retrospectively to at least 1990. In GBD 2021, forecasting to future years was also introduced.

Given their scientific and policy influence, the reliability of these estimates is crucial. However, risk factor epidemiological results may vary widely depending on both genuine differences and biases in different studies and calculations. Establishing both causality and the magnitude of effect is difficult, even for exposures with known health effects.^7^ Capturing long-term outcomes requires follow-up that is difficult to justify in randomized clinical trials, while observational studies are limited by many biases. Unless they address interventions to potentially reduce harm, randomized clinical trials are unethical for harmful factors. Mendelian randomization studies are sensitive to pleiotropy and population stratification, and strong genetic instruments are often lacking.^8^

Overall, evidence on risk factors is often uncertain—particularly for exposures such as diet—that are difficult to quantify and intervene in.^9^ Modeling these factors globally adds another layer of difficulty, given the fragmentary and potentially biased nature of data worldwide. Given the scope of the GBD initiative, understanding the robustness of each assumption and of each piece of data involved in the modeling is challenging. However, given that GBD collaborators update their estimates regularly, the overall stability and consistency of reported estimates can be assessed. The reported (updated and revised) estimates reflect the end product of all changes that happen in the GBD input data, assumptions, and calculations, and these are the numbers that eventually become influential for policy decisions.

A fundamental question is whether these estimates remain relatively steady or change markedly over time. Some changes over time may occur because of genuine decreases (eg, progress in reducing risk factors or finding better interventions to diminish their effects) or genuine increases (eg, wider spread of harmful factors across populations). However, for most risk factors these changes may not be so prominent during the relatively short period from 2010 to 2023, for which 8 different iterations of GBD estimates are available. Unstable estimates for the same risk factor over these iterations may reflect the imperfect data sources and modeling choices, especially when pertaining to historical years. Therefore, by quantifying the instability of GBD estimates empirically, we aimed to indirectly probe their overall reliability. We also sought to understand whether some risk factors have experienced larger fluctuations than others.

## Methods

This was a meta-epidemiological assessment. There is no reporting guideline directly pertinent to this specific design, but we used relevant items from the Preferred Reporting Items for Systematic Reviews and Meta-Analyses (PRISMA) reporting guideline.^10^ Institutional review board approval was not needed because all data came from the published literature. Our analyses are exploratory and no protocol was preregistered. The GBD iterations through 2021 were completed in 2025 and were then updated to include 2023 estimates that became available in October 2025. All code and data are publicly available on GitHub.^11^

### Data Collection and Analytic Datasets

Although age-standardized rates adjust for demographic changes, we relied on overall absolute death and disability-adjusted life-years (DALYs) estimates for each risk factor in this assessment due to their availability and their use in research and policy communication. Data were extracted for all available years from the Institute for Health Metrics and Evaluation repository—manually for years estimated and provided in GBD 2010 to 2017^12,13,14,15,16^ and via the GBD Results Tool for years estimated and provided in GBD 2019 to 2023^2,17,18,19,20,21^ (eTable 1 in Supplement 1). A random sample of 100 manually extracted entries was cross validated by a researcher (A.M.P.), and no discrepancies were identified.

Two analytic datasets were constructed. Dataset 1 comprised death and DALY estimates from each GBD iteration (2010-2017) and overlapping estimates as republished in GBD 2019 to 2023 for years previously estimated (2010, 2013, 2015, 2016, and 2017) as well as the original 2019 to 2023 estimates. Dataset 2 comprised full time-series for dietary risks (1990-2021) estimated in GBD 2019, 2021, and 2023, stratified by sex and geographic location (per World Bank regions), as well as aggregated totals for major causes (noncommunicable diseases, communicable diseases).

### Risk Factor Classification and Harmonization

Risk factors were categorized according to the GBD hierarchical framework (levels 1-3), with level 1 representing broad categories (behavioral, metabolic, and environmental or occupational), level 2 capturing subgroups (eg, tobacco, dietary risks), and level 3 specifying individual risks (eg, diet high in sodium). Terminology was standardized across iterations to ensure comparability (eMethods in Supplement 1). Risk factors with estimates available only in a single iteration were excluded. Level 4 risks were not analyzed for ease of presentation and to focus on the most impactful aggregated risks.

### Statistical Analysis

We first described instability in estimated deaths and DALYs across GBD iterations for the year of analysis (ie, GBD 2010 estimates for 2010 vs GBD 2013 estimates for 2013). Second, we analyzed the differences in the estimates for the same year, including the original estimate (eg, GBD 2015 estimate for 2015) and any subsequent revisions published in later years (eg, GBD 2019 estimate for 2015). The only differentiating factor was the original vs revised iterations of analysis. For both comparisons the instability was summarized using the minimum to maximum range to mean (R:M). We also calculated the coefficient of variation (CV) when the differentiating factor was the GBD iteration. Third, dietary and low physical activity risks that have been subject to debate^22,23,24^ were assessed in greater detail through graphical presentation of absolute estimates and rankings. Lastly, for dietary and low physical activity risks, we compared the GBD 2021 point estimates with the 95% uncertainty intervals (UIs) reported in GBD 2019 (eFigure 3 in Supplement 1); and the GBD 2023 estimates with 95% UIs from GBD 2021 (eFigure 3 in Supplement 1).

In interpreting R:M, there is no previously established rule, but when the range was greater than the mean (R:M >1), it was of concern. With R:M greater than 1.5, we considered instability to be extremely high. For the interpretation of CV, we use standard cutoffs viewing CV greater than 0.2 as high^25^ and CV greater than 0.5 as very high.

## Results

### Analyzed Risk Factors

After harmonization of risk factor naming, 101 unique risk factors were obtained (eMethods in Supplement 1 provides details on harmonization). Of these, 95 were estimated across more than 1 iteration. After excluding 23 level-4 risk factors (primarily occupational risks) plus 5 risk factors related to childhood sexual abuse and bullying, and the combined level 2 risk factor, alcohol and drug use, there were 66 risks factors included in the present analysis. Of these, 1 risk factor was level 0, 3 were level 1, 19 were level 2, and 43 were level 3 (eTable 2 in Supplement 1).

### Level 1 to 3 Risk Factor Estimates Across GBD 2010 to 2023

**Figure 1.**
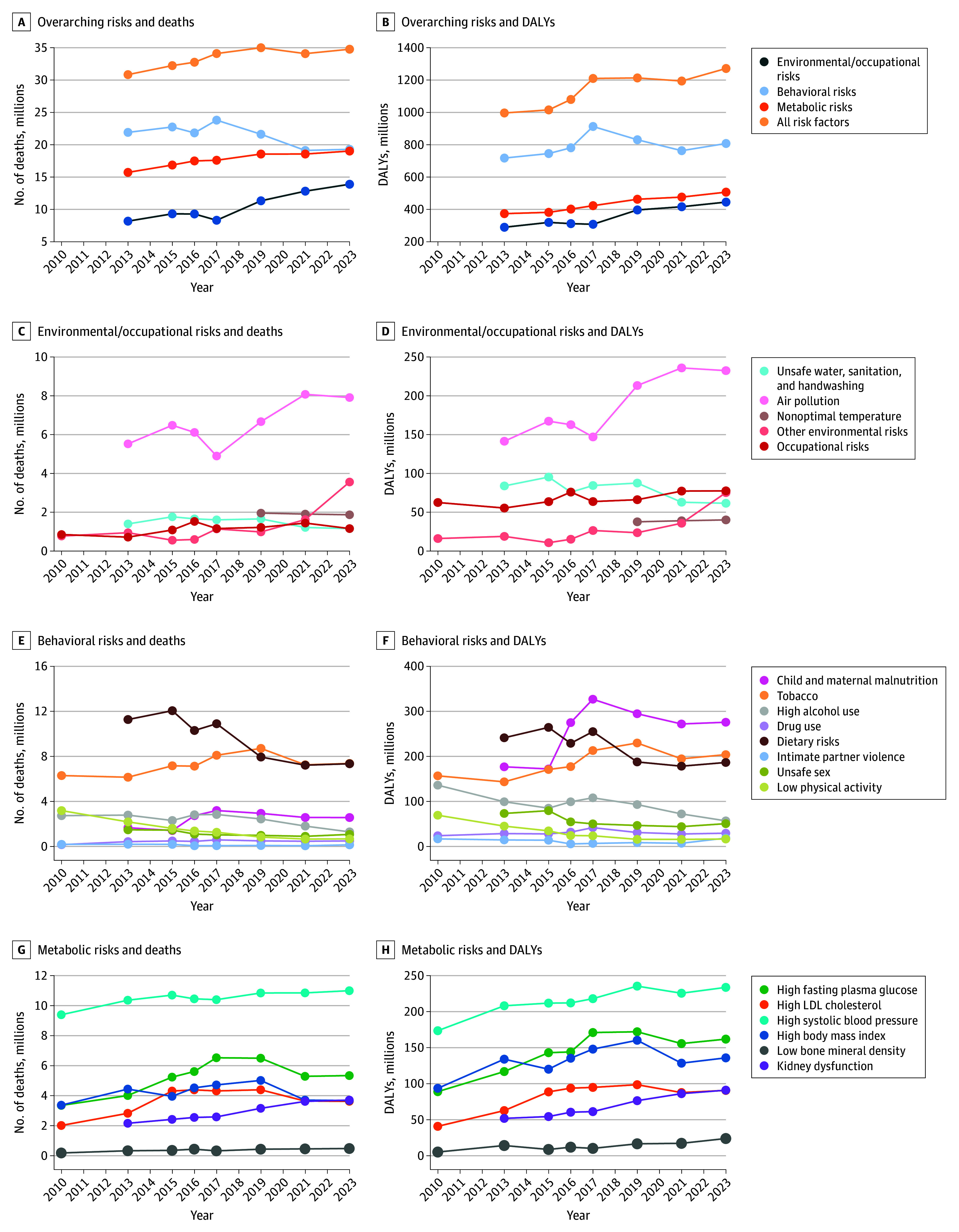
Connected Dot Plot of Estimated Total Deaths and Disability-Adjusted Life-Years (DALYs) Attributable to Level 1 Risk Factors (Overarching Risks) and to Environmental or Occupational, Behavioral, or Metabolic Level 2 Risks Across Global Burden of Disease Iterations LDL indicates low density lipoproteins.

Across successive GBD iterations, estimates of deaths and DALYs attributable to major risk factor categories showed a general increasing trend, particularly for metabolic and environmental or occupational risks, while estimates for behavioral risks display an oscillating behavior (Figure 1). Total deaths attributed to all risk factors rose from 30.8 million in GBD 2013 to 34.8 million in GBD 2023. Deaths attributable to metabolic risks increased steadily over the same period, from 15.7 to 19.0 million, and environmental and occupational risks rose from 8.2 to 13.9 million. In contrast, behavioral risk-related deaths increased initially but returned to earlier levels by 2021. DALY estimates broadly followed similar patterns, with metabolic and environmental risks showing sustained increases, while behavioral risks fluctuated.

At the level 2 risk factor level, deaths attributed to air pollution increased from 4.9 million to 8.1 million in 2017 to 2021, while dietary risks’ estimated deaths declined from 11.3 million to 7.9 million in 2013 to 2019, and deaths associated with low physical activity declined gradually across all iterations from 2.2 million to 670 000.

Among level 3 risk factors (eFigure 4 in Supplement 1), particulate matter pollution death estimates rose from 4.6 million to 7.8 million in 2017 to 2021, lead exposure death estimates increased from 1.5 million to 3.5 million in 2021 to 2023, and smoking death estimates increased from 5.7 million to 7.7 million in 2010 to 2019, and then decreased to 6.2 million.

### Instability of Estimates

Overall, the median (range) of R:M estimates from 2010 to 2023 was 0.8 (0-3.8) for deaths and 0.7 for DALYs (0.1-3.3). When limited to the latest 4 iterations (2017, 2019, 2021, 2023), the median (range) R:M was 0.5 (0-2.9) for deaths and 0.4 (0-2.8) for DALYs. Among level-1 risk categories, the median R:M was 1.0 for deaths and 0.9 for DALYs attributable to behavioral risks, 0.7 for deaths and 0.6 for DALYs attributable to environmental and occupational risks, and 0.5 and 0.6 for deaths and DALYs respectively attributable to metabolic risks. At level 2, the highest R:M values were observed for other environmental risks (2.4 for deaths and 2.3 for DALYs), low physical activity (1.7 for deaths and 1.8 for DALYs), and intimate partner violence (1.1 for deaths and DALYs).

Across the 2010 to 2023 GBD iterations, the risk groups that had the highest proportion of factors with R:M greater than 1 were behavioral risks (17 of 34 [50%] for deaths and 14 of 34 [41%] for DALYs) and environmental and occupational risks (5 of 24 [21%] for deaths and 6 of 24 [25%] for DALYs). The risk factors contributing to the high R:M for the behavioral risks’ death estimates were primarily dietary risks (9 of 16 [56%]), and child and maternal malnutrition (4 of 8 [50%] eTable 3 in Supplement 1). Risk factors with R:M greater than 1.5 for deaths and/or DALYs mainly consisted of behavioral risks, such as dietary risks and child and maternal malnutrition (Table). When we limited the analysis to estimates from the 4 most recent iterations, the proportion of estimates with R:M greater than 1 was 10 of 33 (30%) for behavioral risks’ death estimates (9 of 33 [27%] for DALYs), 2 of 24 (8%) for environmental or occupational risks (2 of 24 [8%] for DALYs) and 0 of 7 for metabolic risks (0 of 7 for DALYs).

**Table.** Global Burden of Disease Estimates of Level 2 Risks With Range to Median Ratio (R:M) Greater Than 1.5 for Deaths or Disability-Adjusted Life-Years (DALYs)

Risk			Deaths			DALYs		
Level 1	Level 2	Level 3						
			Median (range)^a^	R:M	CV	Median (range)^b^	R:M	CV
Behavioral	Child and maternal malnutrition	Iron deficiency	51 (21-199)	2.4	0.8	39 (31-53)	0.5	0.2
		Suboptimal breastfeeding	161 (101-545)	1.7	0.7	14 (9-47)	1.7	0.7
		Vitamin A deficiency	63 (17-233)	2.8	0.9	6 (3-29)	3.1	1
		Zinc deficiency	27 (2-97)	2.7	1	2 (0.1-9)	2.8	1
	Dietary	Diet high in sugar-sweetened beverages	132 (23-300)	2	0.7	5 (1-9)	1.6	0.6
		Diet high in processed meat	306 (130-841)	1.8	0.6	10 (3-21)	1.6	0.6
		Diet high in red meat	73 (25-896)	3.8	1.3	3 (1-24)	3.3	1.2
	Low physical activity	NA	1317 (658-3184)	1.7	0.6	24 (16-69)	1.8	0.6
	Tobacco use	Chewing tobacco	66 (56-251)	1.8	0.9	2 (2-6)	1.7	0.8
Environmental	Occupational	Occupational carcinogens	345 (118-747)	1.7	0.5	8 (3-21)	2.1	0.6
	Other environmental risks	NA	877 (495-3477)	2.5	0.8	19 (9-73)	2.5	0.8
		Lead exposure	965 (558-3560)	2.4	0.8	21 (11-75)	2.3	0.7

Abbreviations: CV, coefficient of variation; NA, not applicable.

^a^ In thousands.

^b^ In millions.

### Original vs Subsequently Revised Estimates for the Same Year

**Figure 2.**
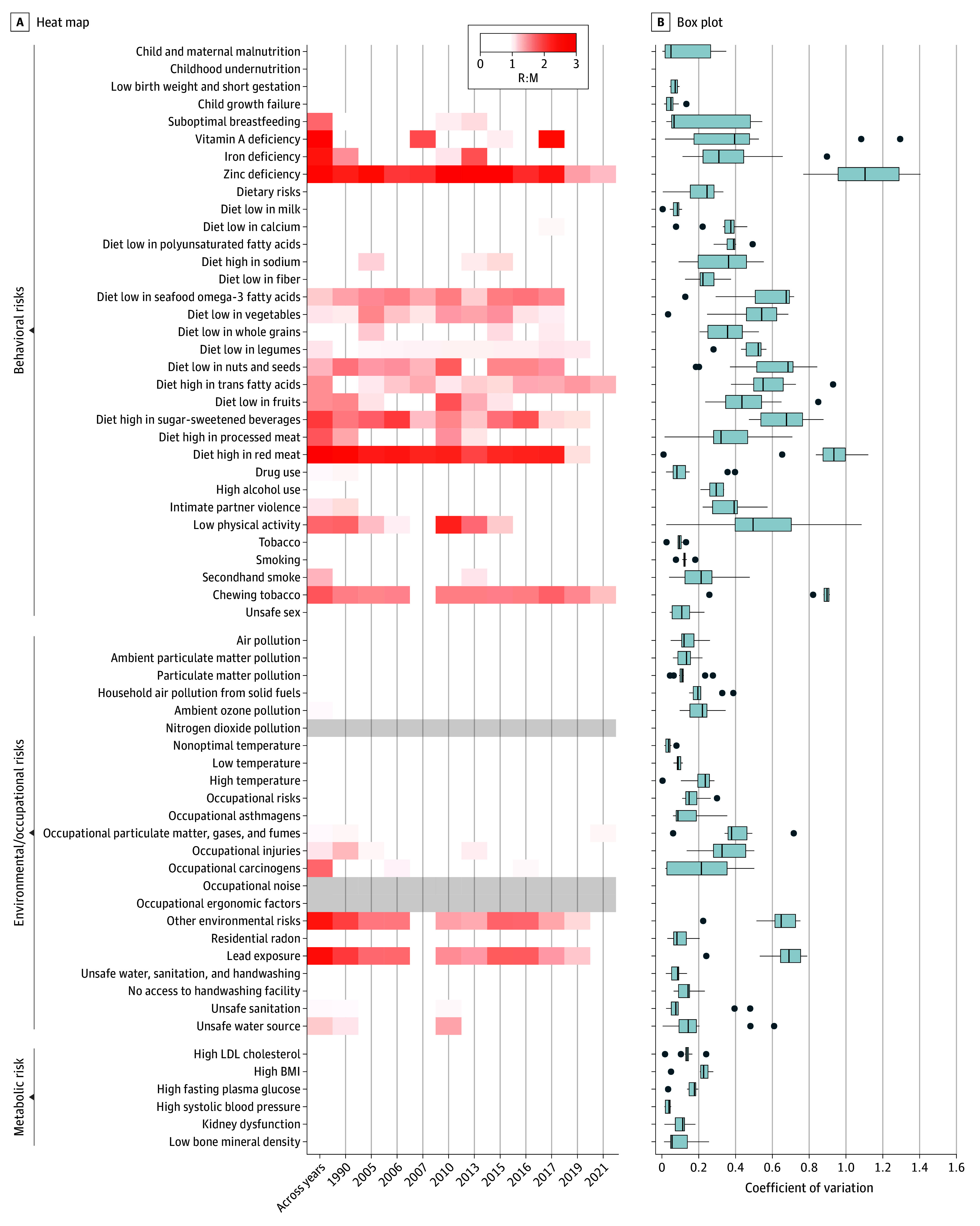
Heat Map of Instability in Death Estimates Using the Range to Mean Ratio (R:M) and Box Plot of the Coefficient of Variation for Global Burden of Disease (GBD) Iterations The first column in the heat map shows instability across index years (eg, GBD 2010 for 2010 through GBD 2023 for 2023), and subsequent columns show instability in matched-year estimates comparing original and revised values for the same calendar year. Gray columns indicate no corresponding death estimates for nitrogen dioxide pollution, occupational noise, and ergonomic factors. In the box plot, the ends of the boxes represent the IQRs; the middle line, the median; and the whiskers, the most extreme data points within ±1.5 × IQR; observations beyond this range are plotted individually. BMI indicates body mass index (calculated as weight in kilograms divided by height in meters squared); LDL, low density lipoprotein.

Across all 66 risk factors and 5100 total estimates from different years in which revisions were performed, median R:M was 0.4 for deaths and 0.4 for DALYs but ranged widely (0-2.9 for deaths and 0-2.9 for DALYs), while the IQRs were narrower (0.2-0.9 for deaths and 0.2-0.8 for DALYs). In addition, 145 of 675 risk factor estimates (21%) had R:M greater than 1 for deaths and 120 of 675 risk factor estimates (18%) had R:M greater than 1 for DALYs. The highest proportions of large instability were seen for behavioral risk factors, with R:M greater than 1 in 111 of 356 (31%) of death estimates and 87 of 356 (24%) of DALY estimates. For environmental and metabolic risks, the same proportions were less than or equal to 10% (eTable 4 in Supplement 1). The level 2 risks that contributed most to the high proportion among the behavioral risks were dietary risks, with a proportion of 74 of 176 (41%) for deaths and 55 of 176 (31%) for DALYs, respectively; child and maternal malnutrition, with a proportion of 19 of 81 (23%) and 14 of 81 (17%) for deaths and DALYs, respectively; and low physical activity with a proportion of 5 of 11 (45%) and 5 of 11 (45%) for deaths and DALYs, respectively. Factors with R:M greater than 1.5 were sugar-sweetened beverages, red meat, fruits, vegetables, nuts and seeds, seafood omega-3 fatty acids, physical activity, iron, zinc and vitamin A deficiency, chewing tobacco, and lead exposure and other environmental factors (Figure 2A).

Furthermore, 336 of 675 (50%) of death estimates had a CV greater than 0.2, and 127 of 675 (19%) had CV greater than 0.5. CVs were mainly elevated among the behavioral and environmental or occupational risks (Figure 2B). Results for DALYs were similar (eFigure 5 in Supplement 1).

**Figure 3.**
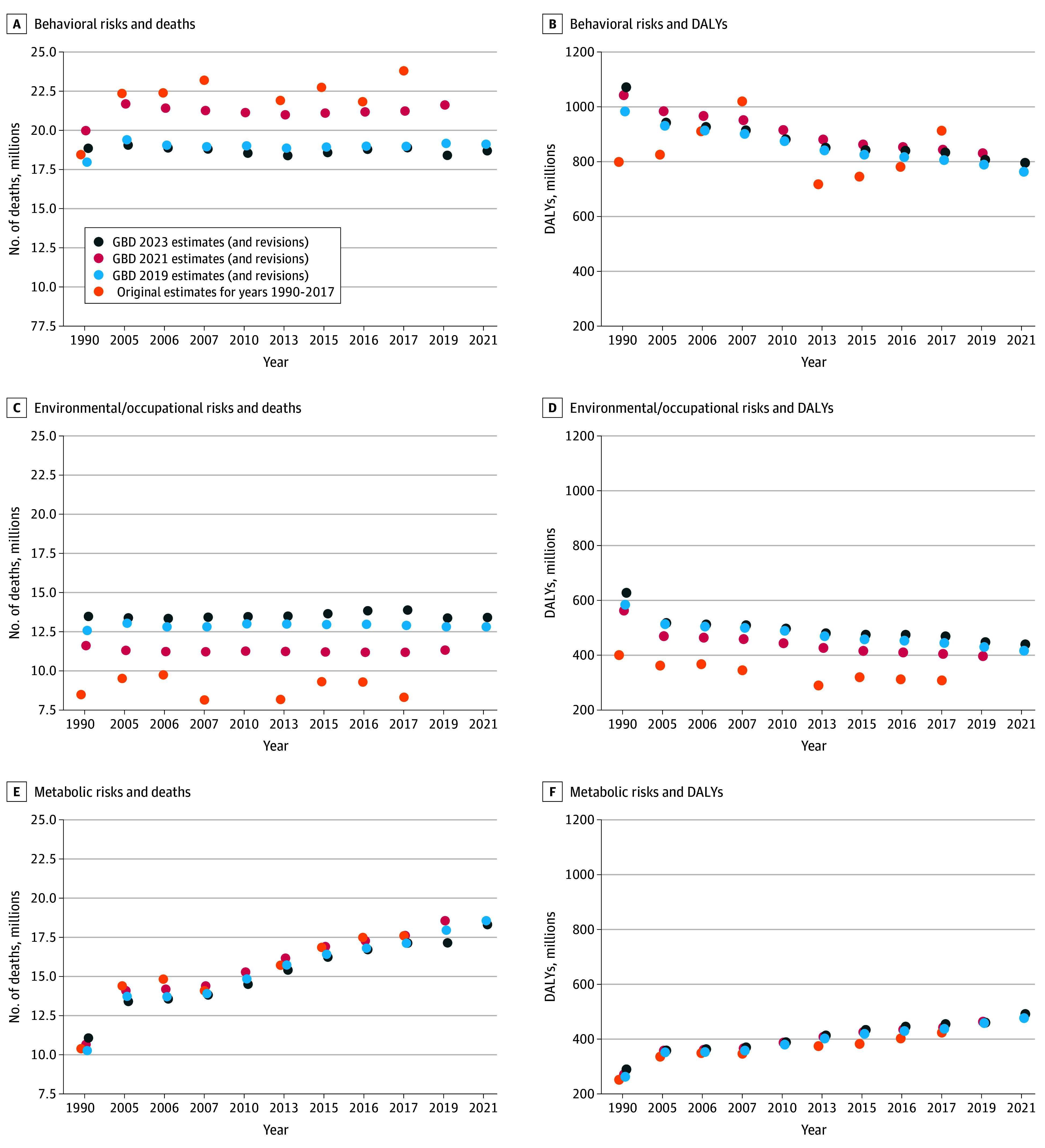
Dot Plots of Matched Risk Year Estimates for Level 1 Risks and Deaths or Disability-Adjusted Life-Years (DALYs), Revised in Global Burden of Disease (GBD) 2023, 2021, and 2019 GBD 2010 did not provide any level 1 risk estimates.

Figure 3 provides a plot of the level-1 risk estimates across GBD iterations and years assessed as an illustration of the deviations for the same risk and year. The deviations were most prominent among environmental or occupational risks and behavioral risks. The same plots for deaths and DALYs of level 2 risks are found in eFigures 6 and 7 in Supplement 1, respectively. In brief, the same discrepancies across dietary risks and child and maternal malnutrition were observed, as well as for air pollution, for both deaths and DALYs.

### Dietary Risk Factors and Low Physical Activity

####  Estimate Trends and Fluctuations

Dietary risk factors and low physical activity are presented in detail in eFigures 8 and 9 in Supplement 1 as an illustrative example for highly debated factors. For example, fluctuations greater than 1 million deaths between iterations were common for diets high in sodium, low in nuts and seeds, and low in whole grains, and for low physical activity. This pattern was also mirrored within the DALY estimates of the dietary risks (eFigure 8 in Supplement 1). Rankings of dietary risk factors also fluctuated substantially across iterations. For example, diet high in processed meat dropped from eighth to thirteenth in attributable deaths between GBD 2017 and GBD 2019, while diet high in red meat rose from sixteenth to fifth for both deaths and DALYs. No dietary risk factor maintained the same rank across more than 3 consecutive GBD iterations, except for diet low in calcium for DALYs (eFigure 9 in Supplement 1).

### Consistency of Estimates Compared to Previous 95% UIs

**Figure 4.**
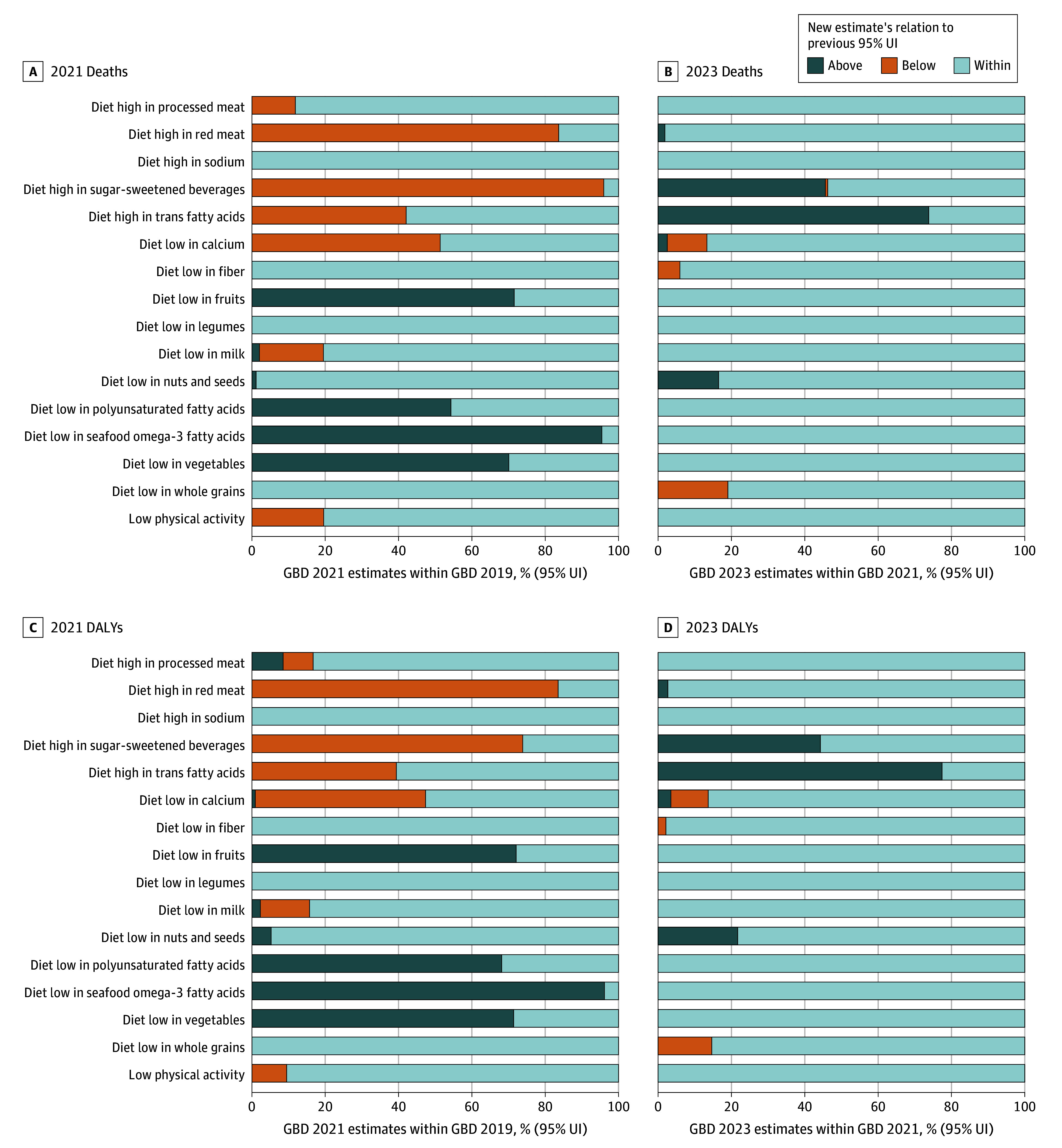
Comparisons of Dietary and Low Physical Activity Risk Estimates in Global Burden of Disease (GBD) 2019 vs 2021 and in GBD 2021 vs 2023 Proportion of GBD 2021 point estimates greater than, less than, and within the corresponding GBD 2019 95% uncertainty interval (UIs) followed by same comparison for GBD 2023 point estimates and GBD 2021 95% UIs.

A comparison of all estimates for dietary risk factors and low physical activity, using sex, region-, and overarching cause-specific estimates, revealed that when comparing the GBD 2021 estimates to the 95% UIs of GBD 2019, approximately half of the revised dietary and low physical activity risk factor estimates fell outside the prior 95% UIs more than 25% of the time. The highest proportion of GBD 2021 death point estimates outside the GBD 2019 95% UI was observed for diet low in vegetables (882 of 1260 [70%]), diet low in fruits (900 of 1260 [72%]), diet high in red meat (1054 of 1260 [84%]), diet low in seafood omega-3 fatty acids (1202 of 1260 [95%]), and diet high in sugar-sweetened beverages (1209 of 1260 [96%]). Similar proportions were observed for DALYs (Figure 4). The comparison of the GBD 2023 point estimate to GBD 2021 95% UI showed highest inconsistency for diet high in trans fatty acids estimates (992 of 1344 [77%]; Figure 4) and sugar-sweetened beverages (921 of 2016 [46%]).

## Discussion

In this analysis of GBD risk factor estimates through 8 iterations spanning from 2010 to 2023, we found substantial instability in mortality and DALY estimates. This was most prominent for behavioral risks such as diet and low physical activity. Even when restricting comparisons to the same calendar years, major differences between original and revised estimates persisted, suggesting that much of the instability between iterations and years reflects methodologic changes rather than true changes in population health. In contrast, metabolic risk estimates were more stable across iterations.

Instability across GBD iterations is unlikely to reflect primarily changes in exposure, incidence, prevalence, and mortality data for the same estimated year. Systematic changes in selection of risk-outcome pairs, such as the adoption of the Burden of Proof Risk Function in GBD 2021, contribute to instability across iterations. For dietary risks and nutritional deficiencies, which are generally supported by low-certainty evidence (0-2 stars), uncertainty in the underlying evidence may also contribute to instability. Variations in how exposure cutoffs are defined further affect estimates; the introduction of empirical cutoffs for dietary risks in 2019 likely affected the estimates. Finally, modifications to the models generating death and disability estimates can produce additional instability, whose pattern is difficult to anticipate due to the complex interdependencies within DisMod and CODEm.

Prior critiques^22,23,24,26^ have emphasized uncertainties in defining dietary exposures, thresholds for low physical activity, and reliance on sparse and low-grade observational data.^27,28,29^ Our results provide empirical evidence that these uncertainties may manifest as unstable estimates across successive GBD publications, which have been undercommunicated in 95% UIs. Furthermore, GBD has been criticized for a more than 2-fold discrepancy between the World Health Organization and GBD death estimates for malaria^4^ and the differences between the Non-Communicable Diseases Risk Factor Collaboration and the GBD estimates of obesity and overweight,^30^ likely due to model specifications. Finally, when a ground truth has been available—such as for “road accidents” in Organisation for Economic Co-operation and Development countries—the GBD overestimated them by 45% compared to the International Road Traffic Accident Database.^31^

### Limitations

Our study has several limitations. First, analyses were limited to published GBD estimates rather than primary data or replication of underlying models. Second, distinguishing true shifts in risk exposure from methodologic artifacts is difficult; however, the degree of divergence between original and revised estimates for the same year suggests that modeling choices may play a major role. Third, early GBD iterations provided few overlapping years, limiting temporal comparisons. Risk factor definitions also evolved over time, although we have tried to minimize the impact of definition changes through harmonization (eMethods in Supplement 1). We should also acknowledge that many GBD estimates are consistent across iterations. Moreover, with the introduction of the Burden of Proof Risk Function^32^ in recent years, 95% UI have increased, communicating higher levels of uncertainty. This may have contributed to making the GBD 2023 and GBD 2021 estimates more commensurate, once uncertainty was accounted for. By focusing on point estimates, some of our results may exaggerate disagreements. For example, R:M calculations do not incorporate the underlying statistical uncertainty of the estimates, just the point estimates. However, the point estimates are most frequently quoted and usually drive policy decisions.

## Conclusions

This meta-epidemiological assessment found that GBD risk factor estimates, especially for behavioral and dietary risks, show marked inconsistency that likely reflects methodologic or data changes rather than true burden shifts. As such, policymakers should routinely consider the uncertainty around GBD estimates. With due caution, they may even take that presented uncertainty as a lower limit of the true uncertainty, whichever is likely greater. Moreover, for factors that have seen large instability over GBD iterations, use of GBD estimates in policy decisions needs to be further tempered. Careful scrutiny of the underlying data and their validity may help inform whether unstable estimates can be of any value in decision-making. The retrospective changes made on prior estimates should not be seen as a signal of degradation in reliability, and the GBD team should be congratulated for striving to improve their methods and data. However, these findings offer a window into how much a policymaker can trust some presented estimates given that they may markedly change retroactively at a future time.

## Notes

Supplement 1. eMethods eFigure 1. Overview of the Global Burden of Disease (GBD) study modeling framework eFigure 2. Timeline of major changes to the GBD modelling approach GBD 2010-2023 eFigure 3. Explanation of comparison of overlapping estimates for GBD 2019 and 2021 eFigure 4. Estimated total deaths and DALYs attributable to Level 3 risk factors across GBD iterations eFigure 5. Variability in death using the range-to-mean ratio and coefficient of variation eFigure 6. Matched risk-year death estimates for level 2 risk factors eFigure 7. Matched risk-year DALY estimates for level 2 risk factors eFigure 8. Death and DALY estimates for the dietary risks across GBD iterations eFigure 9. Changes in rank of dietary risks over time eTable 1. Years analyzed in Global Burden of Disease (GBD) iterations eTable 2. Levels of included risk factors from the Global Burden of Disease (GBD) study eTable 3. Proportion of risks within level 1 and level 2 categories with range/median ratio above 1 eTable 4. Proportion of risks within level 1 and level 2 categories with range/median ratio above 1 for matched risk-year estimates

Supplement 2. Data Sharing Statement
