# Supplementary material for: Instability of Global Burden of Disease Estimates of Deaths and Disability-Adjusted Life-Years From Major Risk Factors: A Meta-Epidemiological Analysis
Source: JAMA Health Forum. 2026 Mar 13;7(3):e260108. doi: 10.1001/jamahealthforum.2026.0108 (PMC12988449; doi:10.1001/jamahealthforum.2026.0108)
Supplement: Supplement 1. — eMethods eFigure 1. Overview of the Global Burden of Disease (GBD) study modeling framework eFigure 2. Timeline of major changes to the GBD modelling approach GBD 2010-2023 eFigure 3. Explanation of comparison of overlapping estimates for GBD 2019 and 2021 eFigure 4. Estimated total deaths and DALYs attributable to Level 3 risk factors across GBD iterations eFigure 5. Variability in death using the range-to-mean ratio and coefficient of variation eFigure 6. Matched risk-year death estimates for level 2 risk factors eFigure 7. Matched risk-year DALY estimates for level 2 risk factors eFigure 8. Death and DALY estimates for the dietary risks across GBD iterations eFigure 9. Changes in rank of dietary risks over time eTable 1. Years analyzed in Global Burden of Disease (GBD) iterations eTable 2. Levels of included risk factors from the Global Burden of Disease (GBD) study eTable 3. Proportion of risks within level 1 and level 2 categories with range/median ratio above 1 eTable 4. Proportion of risks within level 1 and level 2 categories with range/median ratio above 1 for matched risk-year estimates [file jamahealthforum-e260108-s001.pdf]

Supplemental Online Content

Zavalis EA, Pezzullo AM, Ioannidis JPA. Instability of Global Burden of Disease estimates of deaths and disability-adjusted life-years from major risk factors: a meta-epidemiological analysis. *JAMA Health Forum*. Published online March 13, 2026. doi:10.1001/jamahealthforum.2026.0108

eMethods.

eFigure 1. Overview of the Global Burden of Disease (GBD) study modeling framework

eFigure 2. Timeline of major changes to the GBD modelling approach GBD 2010-2023

eFigure 3. Explanation of comparison of overlapping estimates for GBD 2019 and 2021

eFigure 4. Estimated total deaths and DALYs attributable to Level 3 risk factors across GBD iterations

eFigure 5. Variability in death using the range-to-mean ratio and coefficient of variation

eFigure 6. Matched risk-year death estimates for level 2 risk factors

eFigure 7. Matched risk-year DALY estimates for level 2 risk factors

eFigure 8. Death and DALY estimates for the dietary risks across GBD iterations

eFigure 9. Changes in rank of dietary risks over time

eTable 1. Years analyzed in Global Burden of Disease (GBD) iterations

eTable 2. Levels of included risk factors from the Global Burden of Disease (GBD) study

eTable 3. Proportion of risks within level 1 and level 2 categories with range/median ratio above 1

eTable 4. Proportion of risks within level 1 and level 2 categories with range/median ratio above 1 for matched risk-year estimates

This supplemental material has been provided by the authors to give readers additional information about their work.

© 2026 Zavalis EA et al. *JAMA Health Forum*.

eMethods

Harmonization of risk factors across GBD iterations

We harmonized the risk factors' labels to standardize terminology across GBD iterations. All changes involved risk factors that were equivalently defined but inconsistently named between versions. We chose to harmonize high total cholesterol and high LDL cholesterol despite the exposure differing in definition to achieve approximate equivalence of the risks.

Spelling Differences

- **Diet low in fibre** → *Diet low in fiber*
- **Occupational exposure to sulfuric acid** → *Occupational exposure to sulphuric acid*
- **Behavioral risks** → *Behavioural risks*
- **Second hand smoke** → *Second-hand smoke*
- **Secondhand smoke** → *Second-hand smoke*

Specificity in wording (defined the same way)

- **No handwashing with soap** → *No access to handwashing facility*
- **Physical inactivity and low physical activity** → *Low physical activity*
- **Short gestation for birth weight** → *Short gestation*
- **High blood pressure** → *High systolic blood pressure*
- **Alcohol use** → *High alcohol use*
- **Impaired kidney function** → *Kidney dysfunction*
- **Physiological risk factors** → *Metabolic risks*
- **Unsafe water, sanitation and handwashing** → *Unsafe water, sanitation, and handwashing*
- **Unimproved** → *Unsafe* (in water and sanitation contexts)
- **Tobacco smoking** → *Smoking*
- **Occupational risk factors for injuries** → *Occupational injuries*
- **Occupational risk factors** → *Occupational risks*
- **Childhood wasting** → *Child wasting*
- **Childhood stunting** → *Child stunting*
- **Diet low in omega 6 polyunsaturated fatty acids** → *Diet low in polyunsaturated fatty acids*
- **Diet suboptimal in calcium** → *Diet low in calcium*
- **Environmental risks** → *Environmental/occupational risks*
- **Environmental or occupational risks** → *Environmental/occupational risks*

Differences in terms included but same semantic meaning

(Synonymous terms and rewording for terminological alignment)

- **Tobacco smoking (including second-hand smoke)** → *Tobacco*
- **Tobacco smoke** → *Tobacco*
- **Low glomerular filtration rate**→Kidney dysfunction (although proteinuria was added in the definition of kidney dysfunction they correspond to the same level of risk and are non-overlapping as in cholesterol below, low GFR existed between GBD 2013 through GBD 2015 and Kidney dysfunction/Impaired kidney function GBD 2016 through GBD 2021)
- **High total cholesterol** → High LDL cholesterol (although they are not precisely the same the correspond to the same level of risks high total cholesterol existed between in GBD 2010 through GBD 2016 and high LDL cholesterol GBD 2017 through GBD 2021)

Harmonization of levels across GBD iterations

Level 4 risks were removed

- Non-exclusive breastfeeding
- Discontinued breastfeeding
- Childhood underweight
- Occupational exposure to asbestos
- Occupational exposure to arsenic
- Occupational exposure to benzene
- Occupational exposure to beryllium

© 2026 Zavalis EA et al. JAMA Health Forum.

- Occupational exposure to cadmium
- Occupational exposure to chromium
- Occupational exposure to diesel engine exhaust
- Occupational exposure to second-hand smoke
- Occupational exposure to formaldehyde
- Occupational exposure to nickel
- Occupational exposure to polycyclic aromatic hydrocarbons
- Occupational exposure to silica
- Occupational exposure to sulphuric acid
- Occupational exposure to trichloroethylene
- Child wasting
- Child stunting
- Child underweight
- Short gestation
- Low birth weight for gestation
- Low birth weight

Abuse related risk factors with inconsistencies

- Sexual abuse and violence
- Bullying victimization
- Sexual violence against children
- Sexual violence against children and bullying

Alcohol and drug use (Level 2 risk) was removed, and its constituents (namely high alcohol use and drug use) were treated as Level 2 risks instead as in GBD 2019 and GBD 2021.

© 2026 Zavalis EA et al. JAMA Health Forum.

eFigure 1. Overview of the Global Burden of Disease (GBD) study modeling framework

The GBD inputs data into a Bayesian hierarchical model, CODEm (Cause Of Death Estimation model), for deaths and DisMod for non-fatal outcomes. These models estimate deaths, Years of Life Lost (YLL), and Years of Life Lost to Disability (YLD) from input data sources based on information on geography and demography of a specific country. The diseases and injuries are then connected to risk factors through a causal web of risk-outcome pairs evaluated through the Burden of Proof Risk Function (BPRF; until GBD 2019 they used WCRF criteria[1,2], allowing for mediation. Disability Adjusted Life Years (DALYs) are calculated using YLL estimates and disability weights (a measure of the severity of a condition) derived from multi-country surveys conducted in 2010 and 2013, which mainly included respondents from North America and Europe[3]. It is a simplification of the Figure in p.21 of the GBD protocol (https://www.healthdata.org/sites/default/files/files/Projects/GBD/March2020_GBD Protocol_v4.pdf).

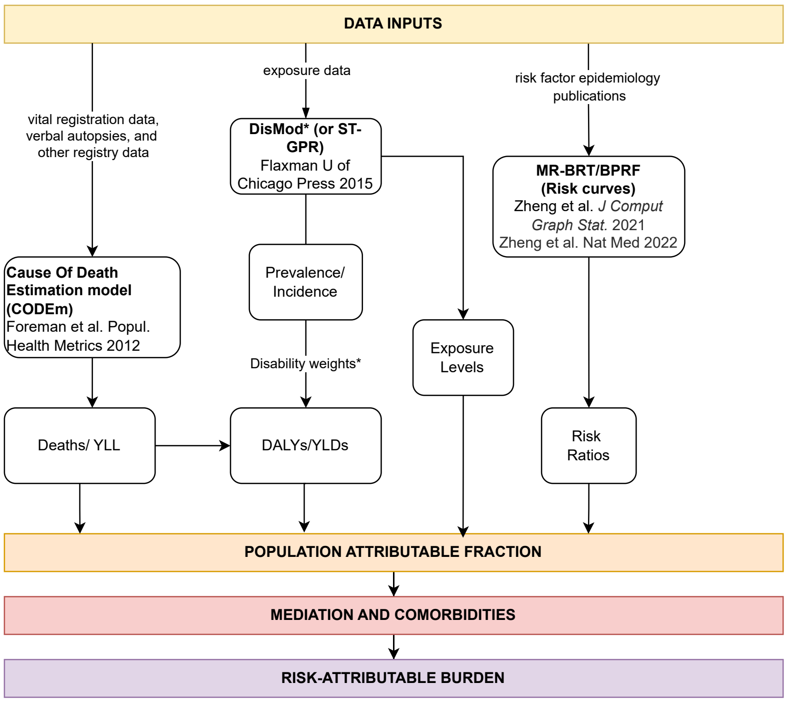

*mainly covering USA, Canada, Hungary, Sweden, Italy and Netherlands (Salomon et al. Lancet 2015)
ST-GPR: spatiotemporal gaussian process regression
BPRF: Burden of Proof Risk Function
MR-BRT: Meta-Regression-Bayesian Regularised Trimmed
DALYs: Disability Adjusted Life Years
YLL: Years of Life Lost
YLD: Years of Life Lost to Disability

1. Zheng P, Afshin A, Biryukov S, et al. The Burden of Proof studies: assessing the evidence of risk. *Nat Med*. 2022;28(10):2038-2044. doi:10.1038/s41591-022-01973-2
2. CUP Global: judging the evidence | World Cancer Research Fund. Accessed January 3, 2026. https://www.wcrf.org/research-policy/global-cancer-update-programme/cup-global-judging-the-evidence/
3. Salomon JA, Haagsma JA, Davis A, et al. Disability weights for the Global Burden of Disease 2013 study. *The Lancet Global Health*. 2015;3(11):e712-e723. doi:10.1016/S2214-109X(15)00069-8

© 2026 Zavalis EA et al. JAMA Health Forum.

eFigure 2. Timeline of major changes to the GBD modelling approach GBD 2010-2023

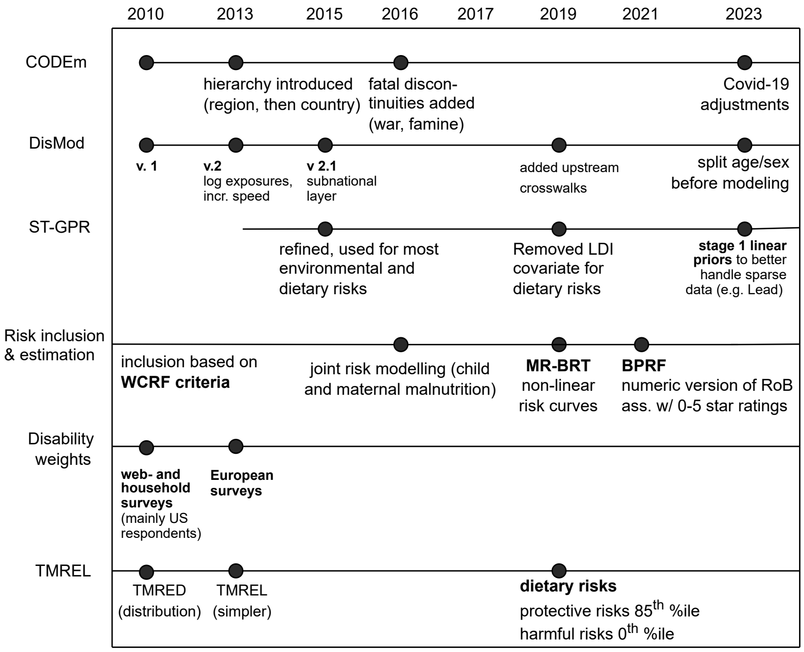

© 2026 Zavalis EA et al. JAMA Health Forum.

eFigure 3. Explanation of comparison of overlapping estimates for GBD 2019 and 2021.

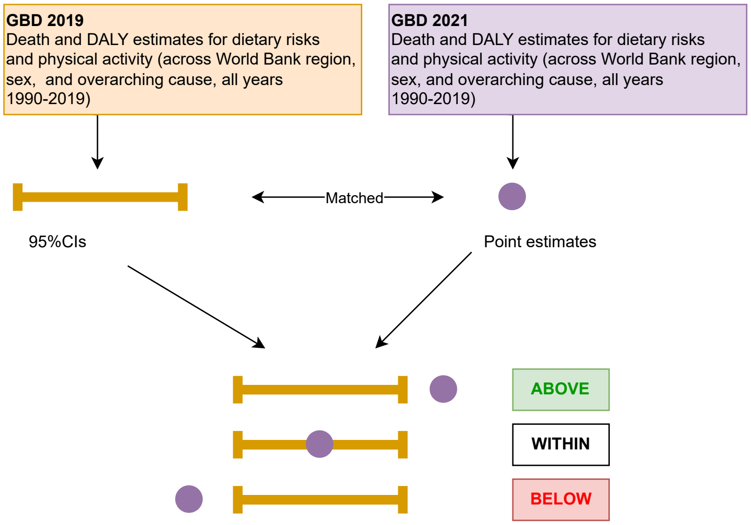

ABOVE means that the GBD 2021 estimate is above the GBD 2019 estimate's 95% confidence interval (CI). WITHIN means that the GBD 2021 estimate is within the GBD 2019 estimate's confidence interval. BELOW means that the GBD 2021 estimate is below the GBD 2019 estimate's confidence interval.

© 2026 Zavalis EA et al. JAMA Health Forum.

eFigure 4. Estimated total deaths and DALYs attributable to Level 3 risk factors across GBD iterations.

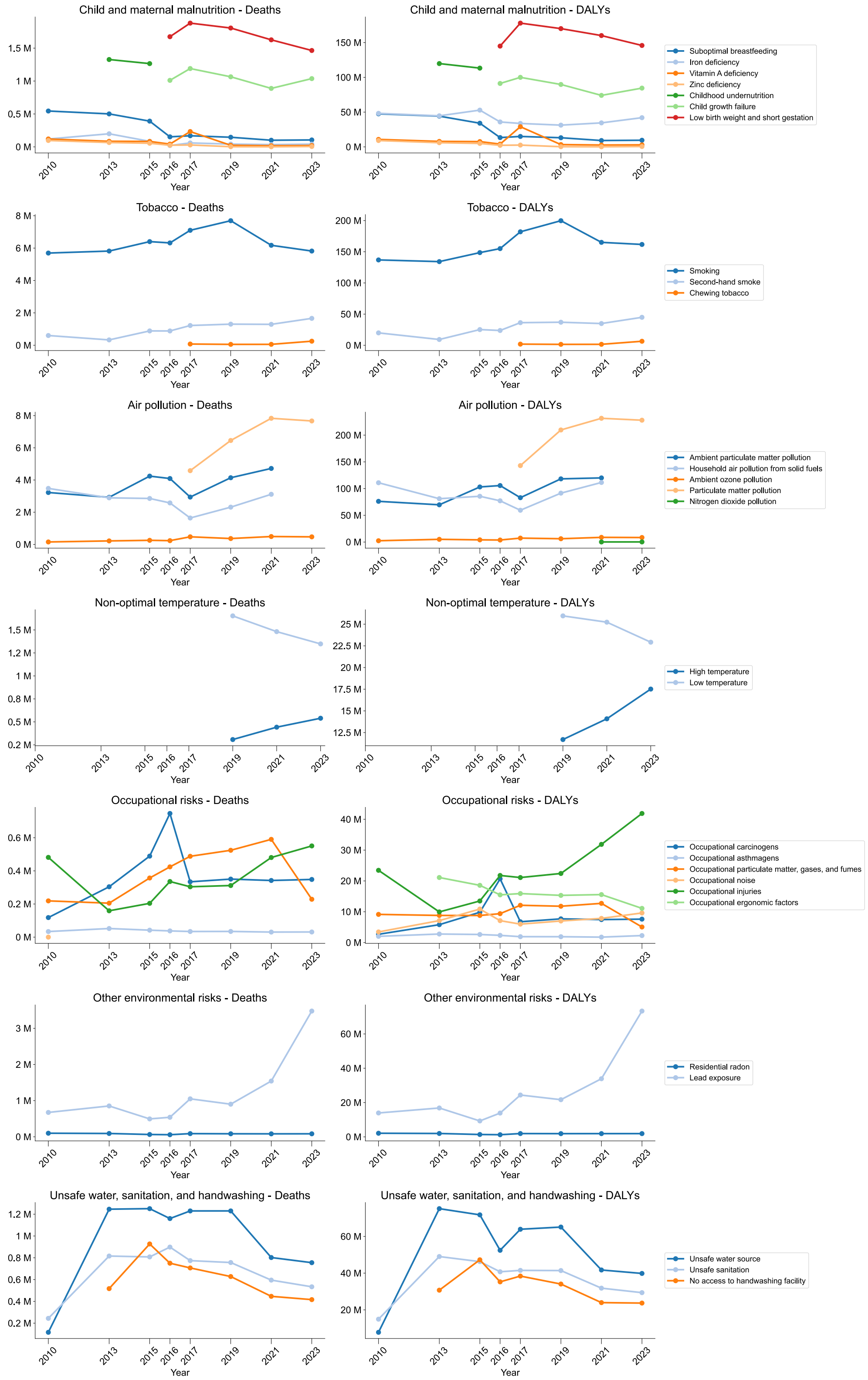

© 2026 Zavalis EA et al. JAMA Health Forum.

eFigure 5. Variability in death using the range-to-mean ratio (A, heatmap) and coefficient of variation (B, boxplot).

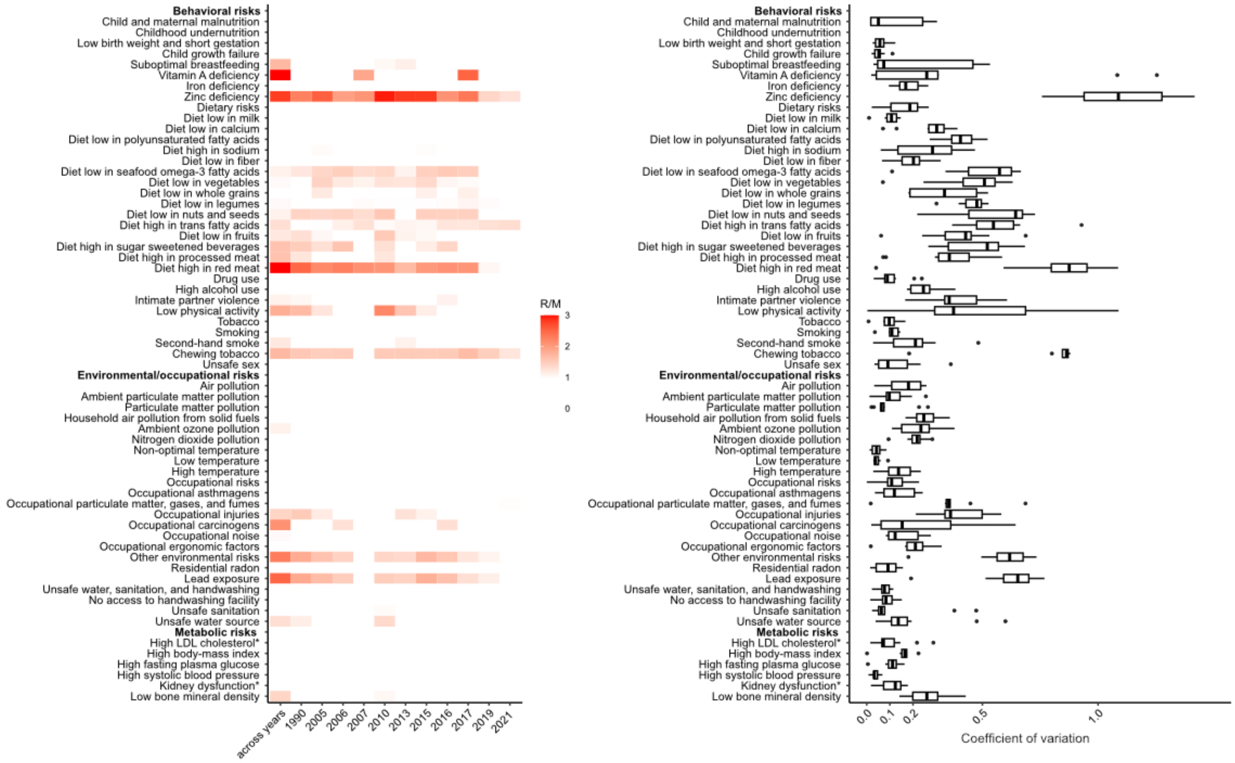

In A) the first column shows variability across index years (e.g., GBD 2010 for 2010 through GBD 2023 for 2023), and subsequent columns show variability in matched-year estimates comparing original and revised values for the same calendar year.

© 2026 Zavalis EA et al. JAMA Health Forum.

eFigure 6. Matched risk-year death estimates for level 2 risk factors.

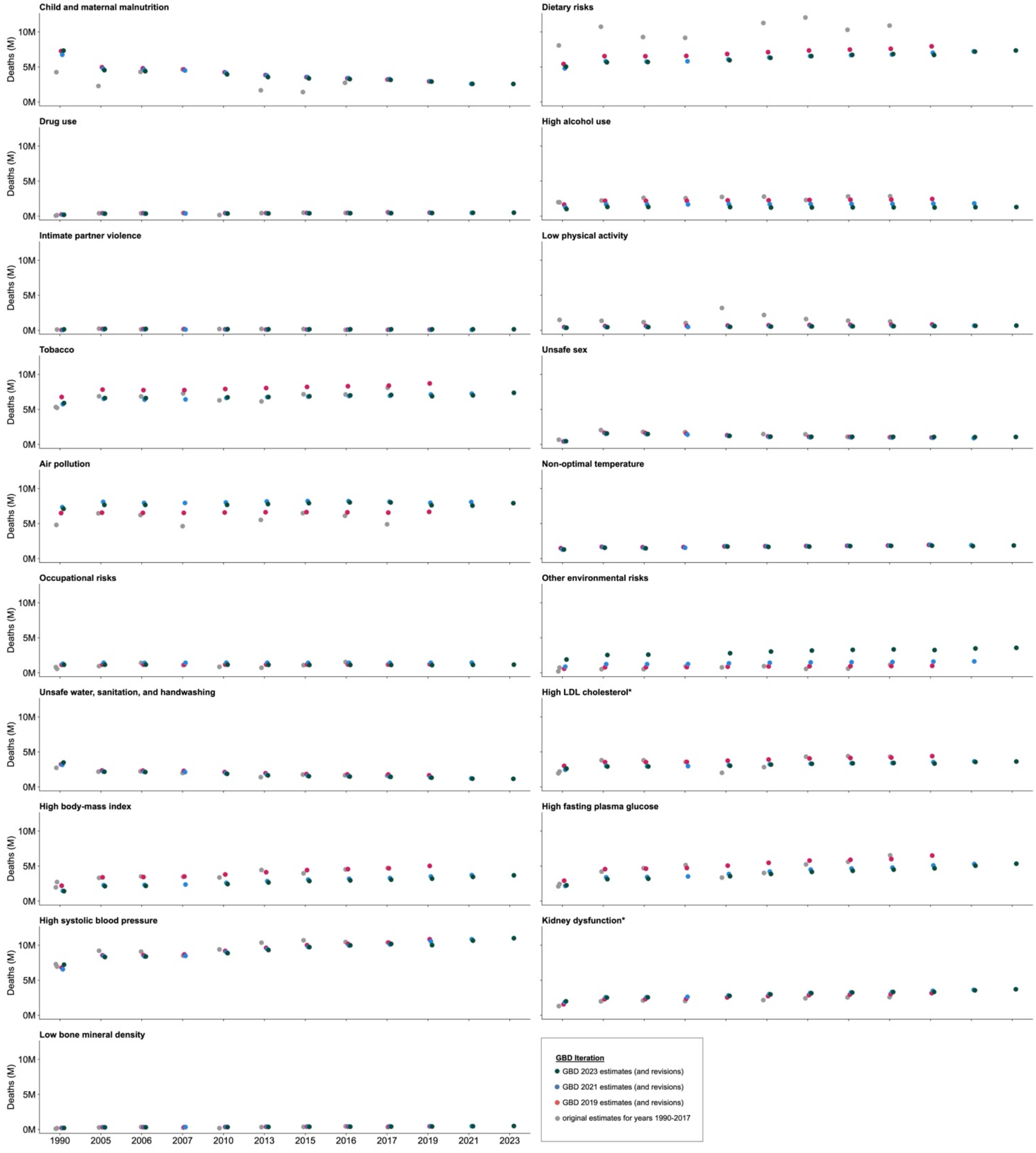

© 2026 Zavalis EA et al. JAMA Health Forum.

eFigure 7. Matched risk-year DALY estimates for level 2 risk factors.

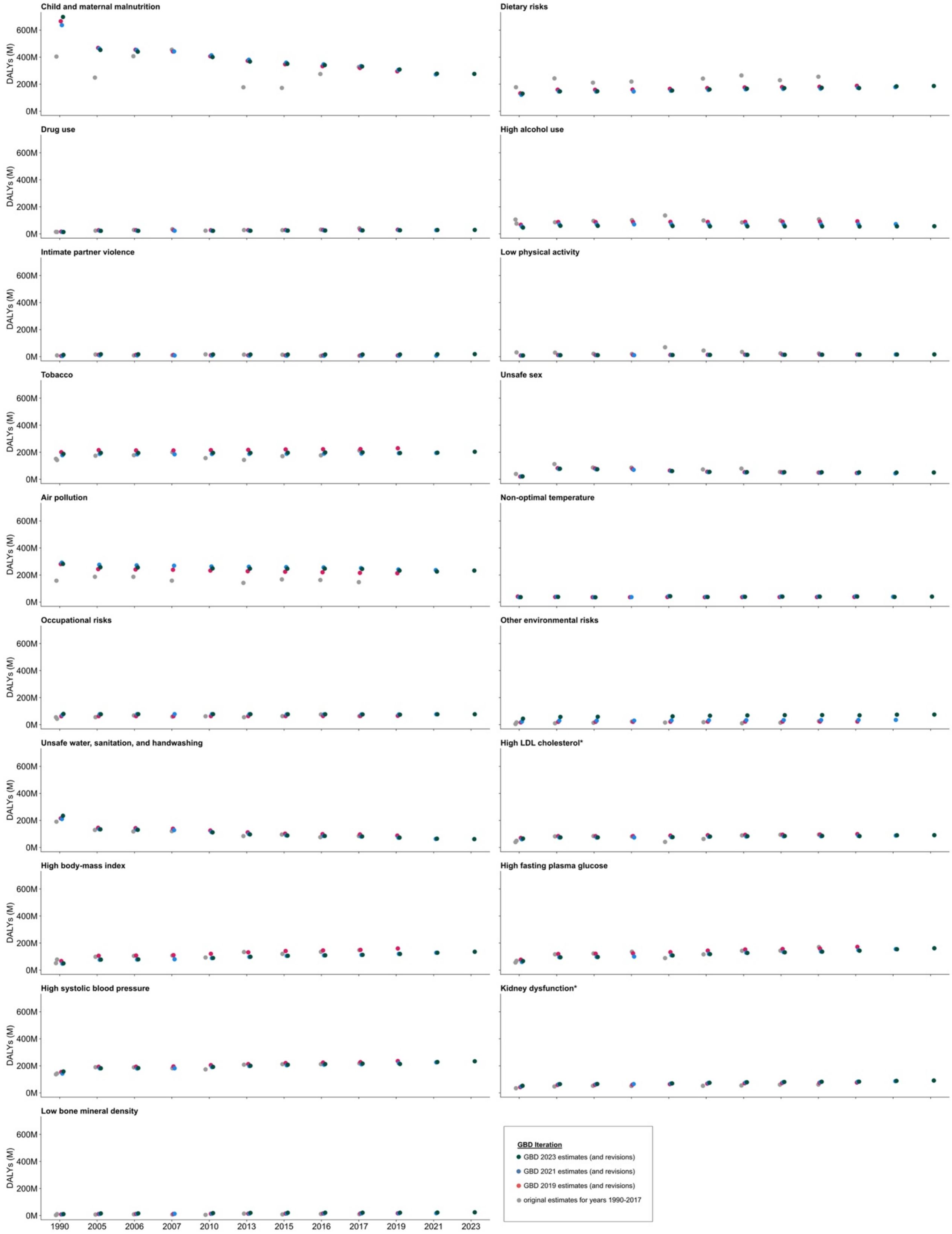

© 2026 Zavalis EA et al. JAMA Health Forum.

eFigure 8. Death (left) and DALY (right) estimates for the dietary risks across GBD iterations.

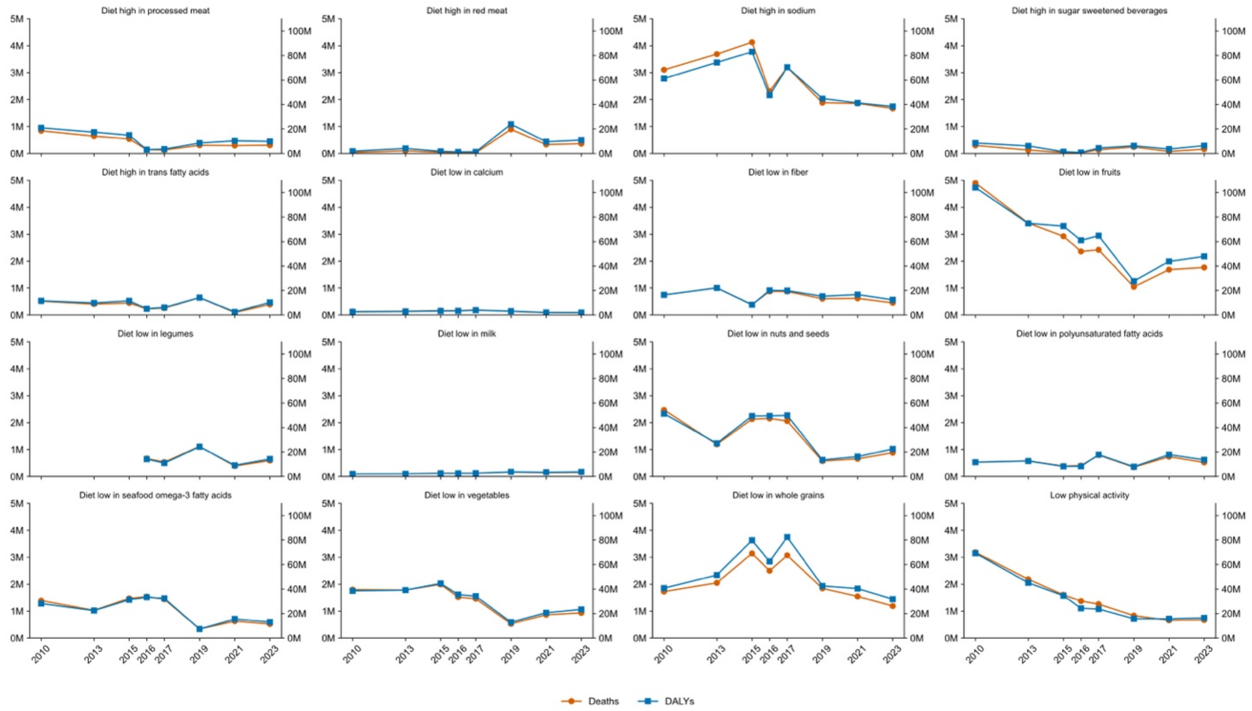

Y-axis is million (M) deaths on the left hand side and DALYs on the right, and the x-axis is GBD years.

© 2026 Zavalis EA et al. JAMA Health Forum.

eFigure 9. Changes in rank of dietary risks over time

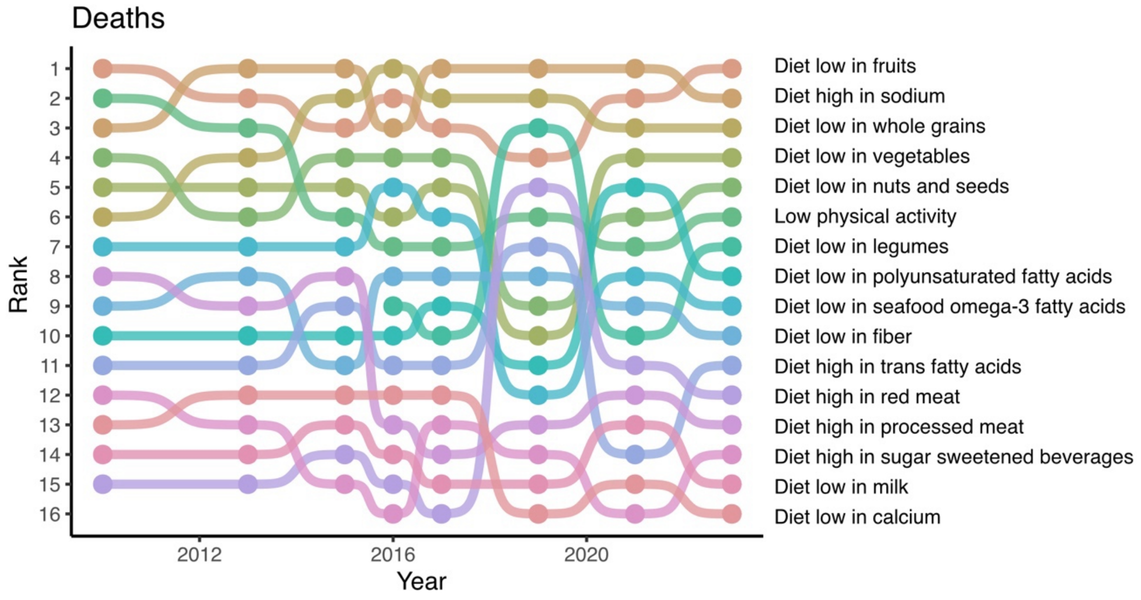

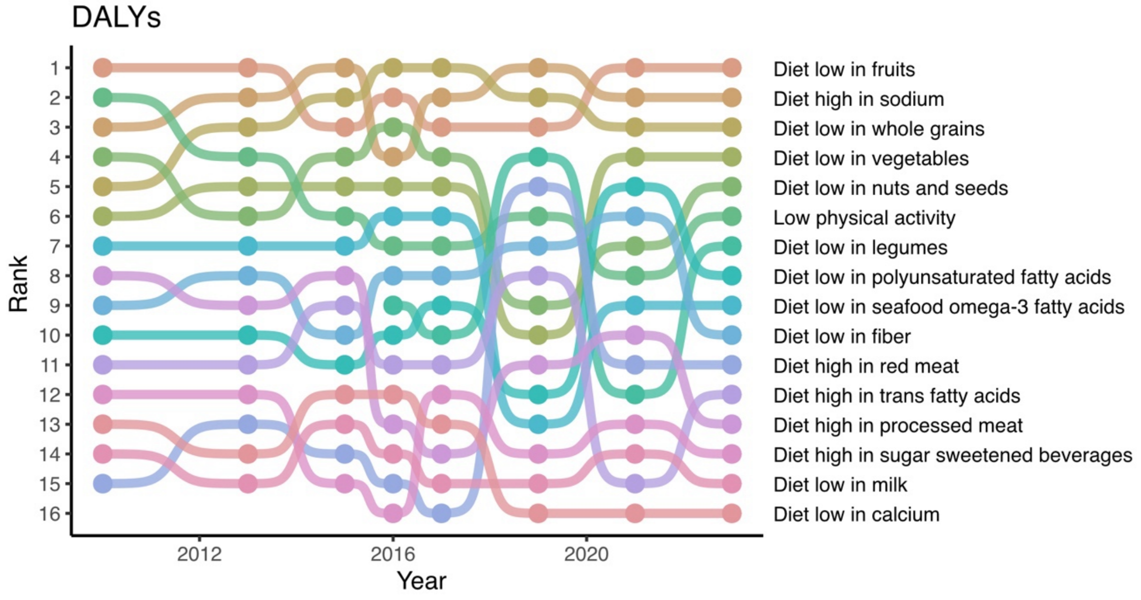

© 2026 Zavalis EA et al. JAMA Health Forum.

eTable 1. Years Analyzed in Global Burden of Disease (GBD) Iterations

*Level 1 risk factors were not estimated in GBD 2010.

GBD iteration	Years estimated in the GBD iteration (utilized in this study)
2010	1990, 2010*
2013	1990, 2013
2015	2005, 2015
2016	2006, 2016
2017	2007, 2017
2019	1990, 2005, 2006, 2007, 2010, 2013, 2015, 2016, 2017, 2019<br>**Dietary risks:** 1980-2019
2021	1990, 2005, 2006, 2007, 2010, 2013, 2015, 2016, 2017, 2019, 2021<br>**Dietary risks:** 1980-2019
2023	1990, 2005, 2006, 2007, 2010, 2013, 2015, 2016, 2017, 2019, 2021<br>**Dietary risks:** 1990-2021

© 2026 Zavalis EA et al. JAMA Health Forum.

eTable 2. Levels of included Risk Factors from the Global Burden of Disease (GBD) Study

**Behavioural risks**	
Child and maternal malnutrition	Suboptimal breastfeeding,<br>Iron deficiency,<br>Vitamin A deficiency,<br>Zinc deficiency,<br>Child and maternal malnutrition,<br>Childhood undernutrition, Child growth failure,<br>Low birth weight and short gestation
Dietary risks	Diet low in fruits, Diet low in vegetables, Diet low in legumes, Diet low in whole grains, Diet low in nuts and seeds, Diet low in fiber, Diet low in milk, Diet low in calcium, Diet low in seafood omega-3 fatty acids, Diet low in polyunsaturated fatty acids, Diet high in red meat, Diet high in processed meat, Diet high in sugar sweetened beverages, Diet high in trans fatty acids, Diet high in sodium
Drug use	
High alcohol use	
Intimate partner violence	
Low physical activity	
Tobacco	Smoking, Second-hand smoke, Chewing tobacco
Unsafe sex	
**Environmental/ occupational risks**	
Air pollution	Ambient particulate matter pollution, Household air pollution from solid fuels, Ambient ozone pollution, Particulate matter pollution, Nitrogen dioxide pollution
Non-optimal temperature	High temperature, Low temperature
Occupational risks	Occupational carcinogens, Occupational asthmagens, Occupational particulate matter, gases, and fumes, Occupational noise, Occupational injuries, Occupational ergonomic factors
Other environmental risks	Lead exposure, Residential radon
Unsafe water, sanitation, and handwashing	Unsafe water source, Unsafe sanitation, No access to handwashing facility
**Metabolic risks**	
High LDL cholesterol*	
High body-mass index	
High fasting plasma glucose	
High systolic blood pressure	
Kidney dysfunction*	
Low bone mineral density	

© 2026 Zavalis EA et al. JAMA Health Forum.

eTable 3. Proportion of risks within level 1 and level 2 categories with range/median ratio above 1.

Level 1	Level 2	Death estimates range/median> 1 (%) (n/N(%))	DALY estimates range/median> 1 (n/N(%))
**Behavioural risks**	**Overall behavioural risks estimate**	17/34 (50)	14/34 (41)
	Child and maternal malnutrition	4/8 (50)	3/8 (38)
	Dietary risks	9/16 (56)	7/16 (44)
	Drug use	0/1 (0)	0/1 (0)
	High alcohol use	0/1 (0)	0/1 (0)
	Intimate partner violence	1/1 (100)	1/1 (100)
	Low physical activity	1/1 (100)	1/1 (100)
	Behavioural risks	0/1 (0)	0/1 (0)
	Tobacco	2/4 (50)	2/4 (50)
	Unsafe sex	0/1 (0)	0/1 (0)
**Environmental and occupational risks**	**Overall risk environmental and occupational estimates**	5/24 (21)	6/24 (25)
	Air pollution	0/6 (0)	1/6 (17)
	Non-optimal temperature	0/3 (0)	0/3 (0)
	Occupational risks	2/7 (29)	2/7 (29)
	Environmental and occupational risks	0/1 (0)	0/1 (0)
	Other environmental risks	2/3 (67)	2/3 (67)
	Unsafe water, sanitation, and handwashing	1/4 (25)	1/4 (25)
**Metabolic risks**	**Overall metabolic risks estimate**	0/7 (0)	1/7 (014)
	High LDL cholesterol*	0/1 (0)	0/1 (0)
	High body-mass index	0/1 (0)	0/1 (0)
	High fasting plasma glucose	0/1 (0)	0/1 (0)
	High systolic blood pressure	0/1 (0)	0/1 (0)
	Kidney dysfunction*	0/1 (0)	0/1 (0)
	Low bone mineral density	0/1 (0)	1/1 (100)
	Metabolic risks	0/1 (0)	0/1 (0)

© 2026 Zavalis EA et al. JAMA Health Forum.

eTable 4. Proportion of risks within level 1 and level 2 categories with range/median ratio above 1 for matched risk-year estimates.

Level 1	Level 2	Death est. R/M>1 (%) (n/N(%))	DALY est/ R/M>1 (n/N(%))
**Behavioural risks**	**Overall behavioural risks estimate**	111/356 (31)	87/356 (24)
	Child and maternal malnutrition	19/81 (23)	14/81 (17)
	Dietary risks	74/176 (42)	55/176 (31)
	Drug use	0/11 (0)	0/11 (0)
	High alcohol use	0/11 (0)	0/11 (0)
	Intimate partner violence	1/11 (9)	1/11 (9)
	Low physical activity	5/11 (45)	5/11 (45)
	Tobacco	12/44 (27)	12/44 (27)
	Unsafe sex	0/11 (0)	0/11 (0)
**Environmental and occupational risks**	**Overall risk environmental and occupational estimates**	24/253 (9)	28/253 (11)
	Air pollution	0/66 (0)	0/66 (0)
	Non-optimal temperature	0/33 (0)	0/33 (0)
	Occupational risks	2/77 (3)	6/77 (8)
	Other environmental risks	20/33 (61)	20/33 (61)
	Unsafe water, sanitation, and handwashing	2/44 (5)	2/44 (5)
**Metabolic risks**	**Overall metabolic risks estimate**	0/66 (0)	0/66 (0)
	High LDL cholesterol*	0/11 (0)	0/11 (0)
	High body-mass index	0/11 (0)	0/11 (0)
	High fasting plasma glucose	0/11 (0)	0/11 (0)
	High systolic blood pressure	0/11 (0)	0/11 (0)
	Kidney dysfunction*	0/11 (0)	0/11 (0)
	Low bone mineral density	0/11 (0)	0/11 (0)

© 2026 Zavalis EA et al. JAMA Health Forum.
