# Supplementary material for: Instability of Global Burden of Disease Estimates of Deaths and Disability-Adjusted Life-Years From Major Risk Factors: A Meta-Epidemiological Analysis
Source: JAMA Health Forum. 2026 Mar 13;7(3):e260108. doi: 10.1001/jamahealthforum.2026.0108 (PMC12988449; doi:10.1001/jamahealthforum.2026.0108)
Supplement: Supplement 2. — Data Sharing Statement [file jamahealthforum-e260108-s002.pdf]

Data Sharing Statement

Zavalis. Instability of Global Burden of Disease Estimates of Deaths and Disability-Adjusted Life-Years From Major Risk Factors. *JAMA Health Forum*. Published March 13, 2026. doi:10.1001/jamahealthforum.2026.0108

Data
Data available: Yes
Data types: Data (not involving human participants)
How to access data: https://github.com/zavalis/pubs_meta/tree/main/gbd%20variability
When available: With publication

Supporting Documents
Document types: Statistical/analytic code
How to access documents: https://github.com/zavalis/pubs_meta/tree/main/gbd%20variability
When available: With publication

Additional Information
Who can access the data: Anyone.
Types of analyses: For any purpose.
Mechanisms of data availability: They are fully available online for anyone to see.
